# Primary osteosarcoma of bladder diverticulum mimicking intradiverticular calculus: a case report

**DOI:** 10.1186/1746-1596-6-37

**Published:** 2011-04-18

**Authors:** Igor Grubišić, Tanja Leniček, Davor Tomas, Tihana Džombeta, Davor Trnski, Igor Tomašković, Božo Krušlin

**Affiliations:** 1 Department of Urology, Sestre milosrdnice University Hospital, Zagreb, Croatia; 2 Ljudevit Jurak Department of Pathology, Sestre milosrdnice University Hospital, Zagreb, Croatia; 3 Department of Pathology, School of Medicine, University of Zagreb, Zagreb, Croatia

## Abstract

There is a well-documented relationship between urinary bladder diverticula and intradiverticular neoplasms. The great majorities of these tumors are urothelial carcinomas, but may also be of glandular or squamous type. Sarcomas occurring within bladder diverticula are exceptionally rare and highly malignant lesions, with only 20 well documented cases published in the literature to date (including carcinosarcomas). We report a case of osteosarcoma of the bladder diverticulum in a 68-year old man, which clinically mimicked intradiverticular calculus. To our knowledge, this is the second case described in the literature to date, and the first in English literature.

## Background

Bladder diverticula mainly present as small, clinically insignificant anomalies. Rarely, probably due to urinary stasis and prolonged exposure to carcinogens, malignancies may arise within them. Analogous to overall distribution of bladder tumors, most intradiverticular tumors also originate from urothelial cells. Adenocarcinomas and squamous cell carcinomas are uncommon and carcinosarcomas and sarcomas are exceptionally rare within diverticula. There are only 20 well documented cases of intradiverticular sarcoma with or without carcinomatous component published in the literature to date [1]. Eleven of them are pure sarcomas, mainly leiomyosarcomas, with isolated cases of osteosarcoma, malignant fibrous histiocytoma, fibrosarcoma and rhabdomyosarcoma. Here, we report a case of primary intradiverticular osteosarcoma which is, to our knowledge, the second case described in the literature to date, and the first in English literature [2].

## Case report

**Figure 1:**
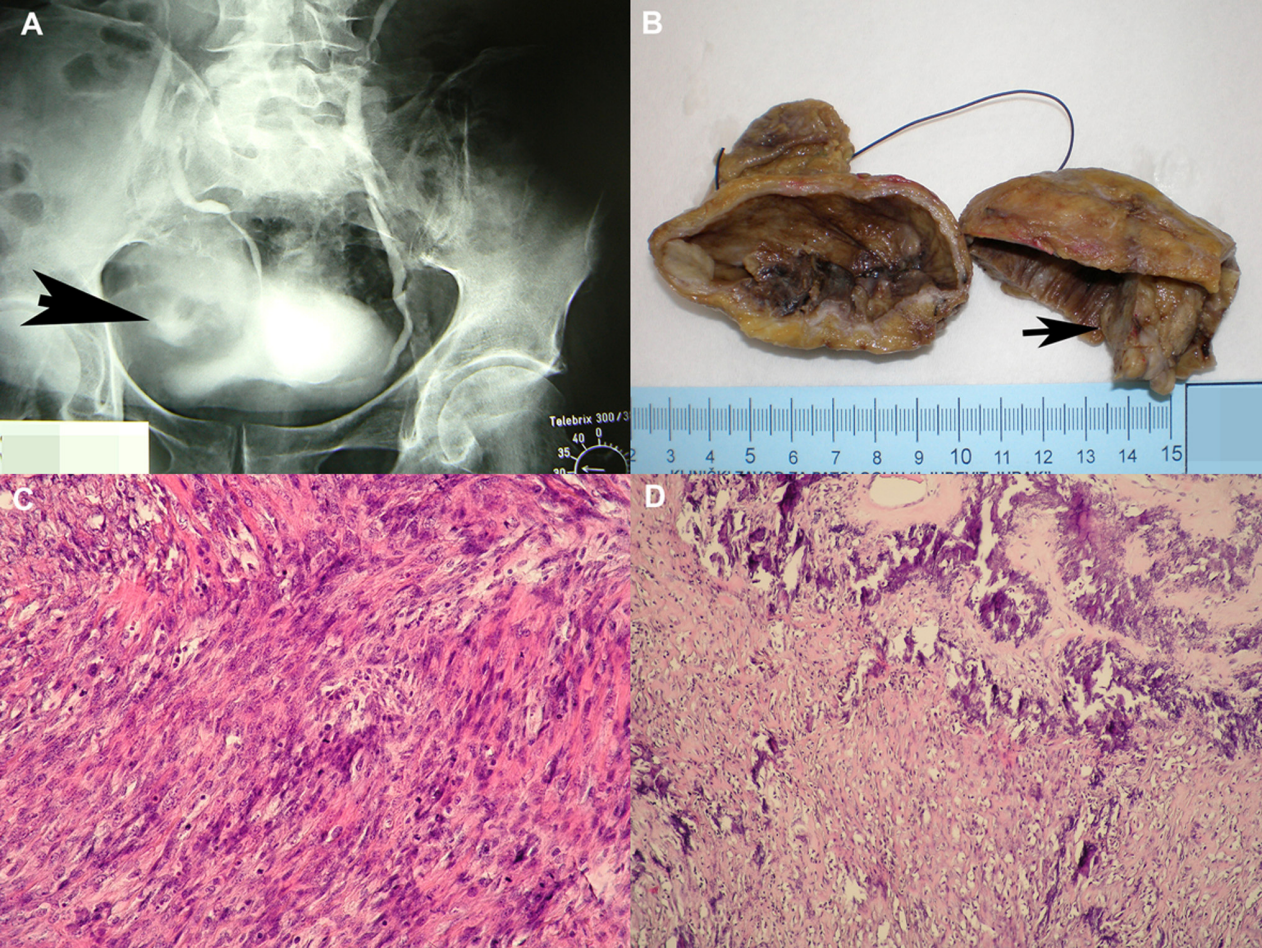
**Radiographic, macroscopic and histological findings**. *A) *Intravenous urography showing large bladder diverticulum, measuring 7 × 7 cm, with 3 cm stone-like component within (arrow). Diverticulum was compressing the bladder and dislocating right ureter towards the central line. *B) *Macroscopically, the bladder diverticulum contained grayish, solid tumor with gritty, firm centre that measured 3.5 cm in the largest diameter (arrow). *C) *The tumor was composed of anaplastic, oval to spindle cells, with prominent mitotic activity, partly rimmed with osteoid (HE, × 200). *D*) The central part of the tumor was composed of mineralized osteoid, deposited as irregular trabeculae with malignant osteocytes (HE, × 100).

A 68-year old man was admitted to our clinic in November 2006 because of an isolated episode of gross hematuria. Intravenous urography, performed a few days later, identified a right-sided bladder diverticulum measuring 5 cm in the largest diameter, with no wall irregularities, or filling defects, which would point to the presence of neoplasm. In October 2007, after a few additional episodes of painless gross hematuria, the patient returned to the clinic and was hospitalized for further diagnostic procedures. Cystoscopy revealed a diverticulum over the right side of the urinary bladder. Intravenous urography showed a large bladder diverticulum measuring 7 × 7 cm with a 3 cm stone within (Figure 1a). The diverticulum was compressing the bladder and dislocating the right ureter towards the central line. Prostate was moderately enlarged. Cystography confirmed the diagnosis of lithiasis of the bladder diverticulum. Diverticulectomy was performed and intraoperative tumorous-like tissue surrounding the stone was found. The specimen was sent to pathology.

Grossly, the bladder diverticulum exhibited tan to brown mucosa with a 3.2 × 3.5 × 1.8 cm greyish, solid tumor with gritty, firm center (Figure 1b). Microscopically, the tumor was composed of atypical, oval to spindle shaped cells with prominent mitotic activity, in some parts rimmed with lacelike osteoid (Figure 1c). In central parts of the tumor, the osteoid was mineralized and deposited as irregular trabeculae with malignant osteocytes within lacunae (Figure 1d). There were 20 slides of the tumor in bioptic material and these were thoroughly examined, but no epithelial component was found. Immunohistochemistry was performed with primary antibodies to cytokeratin (CK), cytokeratin 7 (CK 7), cytokeratin 20 (CK 20), epithelial membrane antigen (EMA), carcinoembryonic antigen (CEA), smooth muscle actin (SMA) and S-100 protein (all DAKO, Denmark). Tumor cells showed negative reaction for CK, CK 7, CK 20, EMA and CEA, which confirmed the absence of epithelial component, specifically the urothelial one. Reaction to SMA was positive, and reaction to S-100 protein was only focally positive. The diagnosis of osteosarcoma was established. The tumor invaded the whole thickness of diverticulum wall with no extension to the perivesical fat. The urothelium adjacent to the tumor showed areas of squamous metaplasia with no cytologic atypia.

Six months postoperatively the patient was alive and well. Extensive clinical examination revealed no signs of other primary tumor.

**Figure 2:**
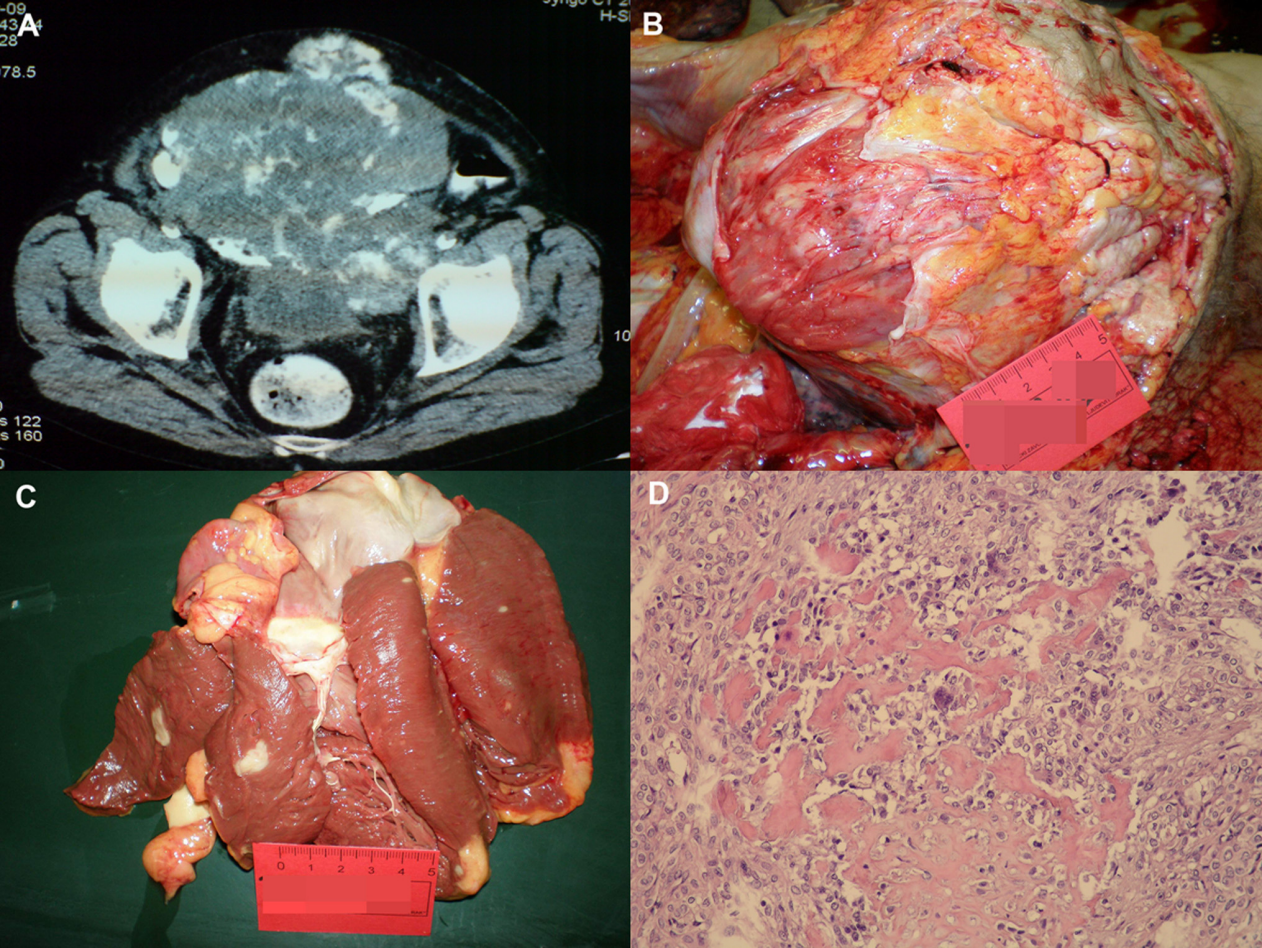
**Radiographic and autopsy findings**. *A*) CT scan showing large intrapelvical tumor. *B) *The macroscopic view of the intrapelvical tumor at autopsy. *C*) Metastatic nodules in myocardium. *D*) Intrapelvical tumor found at autopsy histologically showed picture of osteosarcoma composed of atypical, oval to spindle cells, partly rimmed with osteoid. Multinuclear giant, osteoclast-like tumor cells were also visible (HE, × 200).

In April 2009, the patient was hospitalized again due to macrohematuria. A CT scan revealed extensive tumorous intrapelvical mass (Figure 2a) which was designated as unresectable.

Patient died in July 2009, 2 years and 8 months after the initial symptoms had occurred. The autopsy revealed a large tumor of urinary bladder, measuring 29 × 26 × 18 cm, filling the pelvis, with extension to abdomen (Figure 2b). There were 15 slides of primary tumor in autopsy material and again, no epithelial component was identified. Metastases to lungs, heart and liver were found. There were numerous metastatic nodules in lungs, three nodules in myocardium (Figure 2c) and one nodule in liver. All nodules were histologically confirmed to be metastases of the primary osteosarcoma of the bladder diverticulum (Figure 2d).

## Discussion

Urinary bladder diverticula are outpouchings of bladder mucosa through weakened muscular areas of the bladder wall. They are commonly located in the posterior wall above the trigone, near the ureteral orifices and in the dome at the site of an obliterated urachus. They are mostly acquired, developing secondary due to increased intravesical pressure in patients with urethral or bladder neck obstruction and in patients with neurogenic bladder [3].

There is a well documented relationship between urinary bladder diverticula and intradiverticular neoplasms, with a reported prevalence of 1-10% [4,5]. It seems that urinary stasis produces chronic mucosal irritation and prolonged exposure to urinary carcinogens, which could explain predisposition to malignant transformation of diverticular urothelium [5]. Therefore, the great majority of these tumors are urothelial carcinomas, but may also be glandular or squamous cell carcinomas [6]. Tamas et al. [7] reviewed all bladder diverticula (71 cases) that underwent pathologic sampling in their institution and in half of them (51%) found neoplastic changes that ranged from in situ lesions to invasive carcinoma. The majority of invasive lesions were urothelial carcinomas (70%) and the remaining cases included small cell carcinoma, squamous cell carcinoma, sarcomatoid carcinoma and adenocarcinoma. The incidence of intradiverticular neoplastic changes was significantly higher than in other studies, probably because the diverticula were resected due to abnormal cistoscopy findings while benign-appearing diverticula found on imaging and cystoscopy weren't included [7]. Cases of small cell carcinoma, paraganglioma, malignant fibrous histiocytoma and even metastasis of gastric adenocarcinoma are also described within the bladder diverticulum [8-10]. However, sarcomas occurring within bladder diverticula are exceptionally rare and highly malignant lesions. The first comprehensive study on this topic was done in 2004, by Cheng et al. [1] who reviewed 18 cases of carcinosarcoma and pure sarcoma of bladder diverticulum and described one additional case of carcinosarcoma [1]. We summarized the described cases of intradiverticular sarcomas, with or without carcinomatous component, including our case of osteosarcoma (Table 1) [1,9,11-13]. In all described cases patients were male, with the age range from 47 to 87 years (average 65.8 years). These cases are available at PubMed. Lack or thinness of muscular layer of the diverticulum predisposes to early peridiverticular invasion. Consequently, intradiverticular tumors tend to be more advanced in stage and, apart from some exceptions, the outcome of these patients is poor despite radical therapy. Among these 19 cases, there was only one case of intradiverticular osteosarcoma published in Italian by Sarno et al. [2]. Authors reported a case of 77-year old man with hypertrophic prostate, who was also hospitalized because of macrohematuria. These authors focused more on the tumor's radiological presentation and the importance of preoperative imaging [2].

**Table 1:** Cases of intradiverticular sarcomas with or without carcinomatous component reported in the literature to date

	Authors	Year published	Sex	Age	Tumor type
1	Grubišić I et al	/	M	68	Osteosarcoma

2	Tsujita Y et al	2009	M	68	Leiomyosarcoma

3	Cheng CW et al	2004	M	71	Carcinosarcoma

4	Omerglu A et al	2002	M	65	UC + sarcoma

5	Bigotti G et al	2001	M	87	SCC + sarcoma resembling MFH

6	Escandon AS et al	2000	M	69	SCC + sarcoma

7	Garcia Figuerias R et al	2000	M	69	SCC + sarcoma

8	Hara S et al	1999	M	71	UC + SCC + leiomyosarcoma

9	Takei K et al	1995	M	77	Leiomyosarcoma

10	Begara Morillas F et al	1995	M	/	Leiomyosarcoma

11	Nuwahid F et al	1994	M	47	UC + chondrosarcoma

12	Sarno A et al	1991	M	/	Osteosarcoma

13	Koizumi H et al	1987	M	56	Carcinosarcoma

14	McCormick SR et al	1985	/	/	MFH

15	Doctor VM, Karanjavala DK	1975	M	68	Fibrosarcoma

16	Ostroff EB et al	1973	M	61	Carcinosarcoma

17	Ostroff EB et al	1973	M	57	Sarcoma

18	Murdzhiev A, Bozhilov I	1971	M	/	Leiomyosarcoma

19	Bolten B	1967	M	72	Sarcoma

20	Richany SF	1965	/	/	Rhabdomyosarcoma

21	De Miguel S, Gutierrez Sanz E	1960	/	/	Sarcoma

* UC - urothelial carcinoma; SCC - squamous cell carcinoma; MFH - malignant fibrous histiocytoma

In the present case, the tumor mimicked diverticular lithiasis on radiological examination, but histologically it was composed of irregular, lacelike osteoid and closely packed anaplastic, round to spindle cells with prominent mitotic activity. More towards the center of the tumor, the osteoid was mineralized. These findings, which clearly describe the image of osteosarcoma, should be differentiated from other bone-forming tumors, like carcinosarcoma, urothelial carcinoma with osseous metaplasia and, theoretically, metastasis of osteosarcoma of other primary sites [14,15]. The bone in the mesenchymal component of carcinosarcoma, like that in osteosarcoma, is neoplastic while that in the urothelial carcinoma with osseus metaplasia is benign-appearing. However, these can be easily distinguished from the osteocarcoma by the presence of neoplastic epithelial elements. In the present case, despite multiple sectioning, no epithelial components were found within the tumor, which was confirmed by negative immunohystochemical reactions for CK, CK 7, CK 20, EMA and CEA. The diagnosis of osteosarcoma was confirmed at autopsy.

## Conclusion

In most cases, bladder diverticula are small and asymptomatic, but occasionally malignant transformation may occur within them. Overall incidence of sarcomas is the lowest one among urinary bladder tumors. Our case of primary intradiverticular osteosarcoma is the first such case reported in English literature. We find it very important to make a thorough investigation in patients with symptomatic diverticula because once a malignant transformation had occured, there is a predisposition for rapid spread of the tumor due to lack of muscular layer in diverticular wall.

## Consent

Written informed consent was obtained from the patient's family for publication of this case report and accompanying images. A copy of the written consent is available for review by the Editor-in-Chief of this journal.

## Competing interests

The authors declare that they have no competing interests.

## Authors' contributions

IG supplied relevant clinical information about the patient, drafted the manuscript and acquired radiographs. TL acquired photomicrographs, was involved in the histopathological evaluation and drafted the manuscript. DT and BK participated in the histopathological evaluation and have been involved in revising the manuscript critically. TD was involved in literature search and drafting the manuscript. DT and IT supplied relevant clinical information about the patient and were involved in preparing the material. All authors have read and approved the final manuscript.
